# Publisher Correction: Persistent meanders and eddies lead to quasi‑steady Lagrangian transport patterns in a weak western boundary current

**DOI:** 10.1038/s41598-021-89612-7

**Published:** 2021-05-06

**Authors:** M. B. Gouveia, R. Duran, J. A. Lorenzzetti, A. T. Assireu, R. Toste, L. P. de F. Assad, D. F. M. Gherardi

**Affiliations:** 1 Division of Remote Sensing, National Institute for Space Research, São José Dos Campos, 12227-010 Brazil; 2 National Energy Technology Laboratory, Albany, OR 97321 USA; 3 Theiss Research, La Jolla, CA 92037 USA; 4 Natural Resources Institute, Federal University of Itajubá, Itajubá, 37500‑015 Brazil; 5 Laboratory for Computational Methods in Engineering, COPPE/UFRJ, Rio de Janeiro, 21941‑907 Brazil

Correction to: *Scientific Reports* https://doi.org/10.1038/s41598-020-79386-9, published online 12 January 2021

This Article contains errors in the Supplementary Information, where Supplementary Figure files are missing. These files are provided below.

## Supplementary information

Supplementary Figure 1.

Supplementary Figure 2.

Supplementary Figure 3.

Supplementary Figure 4.

Supplementary Figure 5.

Supplementary Figure 6.

Supplementary Figure 7.

Supplementary Figure 8.

Supplementary Figure 9.

Supplementary Figure 10.

Supplementary Figure 11.

Supplementary Figure 12.

Supplementary Figure 13.

