# Supplementary material for: Persistent meanders and eddies lead to quasi-steady Lagrangian transport patterns in a weak western boundary current
Source: Sci Rep. 2021 Jan 12;11:497. doi: 10.1038/s41598-020-79386-9 (PMC7804456; doi:10.1038/s41598-020-79386-9)
Supplement: Supplementary file 1 — Supplementary Information [file 41598_2020_79386_MOESM1_ESM.pdf]

Supplementary Information for "Persistent meanders and eddies lead to quasi-steady Lagrangian transport patterns in a weak western boundary current

M. B. Gouveia[1,*], R. Duran[2,3], J. A. Lorenzzetti[1], A. T. Assireu[4], R. Toste[5], L. P. de F. Assad[5], and D. F. M. Gherardi[1]

[1]Division of Remote Sensing, Brazilian National Institute for Space Research, São José dos Campos, 12227-010, Brazil
[2]National Energy Technology Laboratory, Albany, OR 97321, USA
[3]Theiss Research, La Jolla, CA, 92037, USA
[4]Natural Resources Institute, Federal University of Itajubá, Itajubá, 37500-015, Brazil
[5]Laboratory for Computational Methods in Engineering, COPPE/UFRJ, Rio de Janeiro, 21941-907, Brazil
[*]mainarabg@gmail.com

Contents

- Appendix A. Drifter trajectories
- Appendix B. Monthly-mean model surface velocity
- Appendix C. Seasonal trajectories
- Appendix D. Persistent Lagrangian transport patterns
- Appendix E. Satellite sea-surface temperature derived from MUR
- Appendix F. Model and satellite seasonal means
- Appendix G. Eulerian and Lagrangian divergence

1 Drifter trajectories

Figure S 1. Trajectories of drifters with drogues (Surface Velocity Project; SVP) over annual $c\rho$. a) 43621, b) 45969, c) 46072, and d) 71895. The 200- and 2000-m depth contours are represented by thin black lines respectively. Drifter numbers are located on the bottom right of each picture frame. Red dots are the initial position of each trajectories (purple lines) and blue dots are the final position. Created with Matlab R2018B (www.mathworks.com).

Figure S 2. Trajectories of synthetic drifters over annual $c\rho$. On the left side are the trajectories of the 30 synthetic drifters launched on December 1, 2013 whose destinations are on February 28, 2014. On the right side are the trajectories of the 30 synthetic drifters launched on June 1, 2006 whose destination on September 30, 2006. The 200- and 2000-m depth contours are represented by thin black lines respectively. Red dots are the initial position of each trajectories (purple lines) and blue dots are the final position. Created with Matlab R2018B (www.mathworks.com).

Figure S 3. a) SVP 71891 drifter over annual $c\rho$. b) trajectories of the 30 synthetic drifters launched on December 1, 2013 whose destination on February 28, 2014. c) trajectories of the 30 synthetic drifters launched on June 1, 2006 whose destination on September 30, 2006. The 200- and 2000-m depth contours are represented by thin black lines respectively. Red dots are the initial position of each trajectories (purple lines) and blue dots are the final position. Created with Matlab R2018B (www.mathworks.com).

2 Monthly-mean model surface velocity

File name: S_04_cRHO_Quiver.gif

Figure S 4. Monthly $c\rho$(in logarithmic scale) over respective monthly-mean model surface velocity (black arrows). The 200- and 2000-m depth contours are represented by thin black lines respectively. Created with Matlab R2018B (www.mathworks.com).

File name: S_05_SSH_Quiver_cLCSs.gif

Figure S 5. Monthly cLCS (white lines) over respective monthly-mean model surface velocity (black arrows) and monthly-mean model SSH (color shading). The 200- and 2000-m depth contours are represented by thin black lines respectively. Created with Matlab R2018B (www.mathworks.com).

3 Seasonal trajectories

Figure S 6. All Summer trajectories (Jan - Feb - Mar) over February - $c\rho$(a). All Autumn trajectories (Ap - May - Jun) over May - $c\rho$(b). All Winter trajectories (Jul - Aug - Sep) over August - $c\rho$(b). All Spring trajectories (Oct - Nov - Dec) over November - $c\rho$(d). The 200- and 2000-m depth contours are represented by thin black lines respectively. Created with Matlab R2018B (www.mathworks.com).

4 Persistent Lagrangian transport patterns

Figure S 7. Monthly cLCS (squeezelines) of the study area are colored according to monthly $c\rho$. Depth contours of 200- and 2000-m are indicated by thin black lines. Created with Matlab R2018B (www.mathworks.com).

5 Satellite sea-surface temperature derived from MUR

Figure S 8. SST from MUR sensor showing the eddies of Vitória, São Tomé, Cabo Frio and Cabo de Santa Marta. Images correspond to a) April 22, 2006; b) August 08, 2013; c) June 10, 2007; d) September 10, 2010. Depth contours of 200- and 2000-m are indicated by thin black lines. Note that color bars are different for each image. Created with Matlab R2018B (www.mathworks.com).

6 Model and satellite seasonal means

Figure S 9. Seasonal average of model SSH (color shading) for a) Summer (Jan - Feb - Mar) over seasonal average model surface velocity (black arrows) and February cLCS (white lines). In b) is the seasonal average model SSH (color shading) for Winter (Jul - Aug - Sep) over seasonal average model surface velocity (black arrows) and August cLCS (white lines). Depth contours of 200- and 2000-m are indicated by thin black lines. Created with Matlab R2018B (www.mathworks.com).

Figure S 10. Seasonal average of MUR-SST (color shading) for a) Summer (Jan - Feb - Mar) over seasonal average model surface velocity (black arrows) and February cLCS (white lines). In b) seasonal average MUR-SST (color shading) for Winter (Jul - Aug - Sep) over seasonal average model surface velocity (black arrows) and August cLCS (white lines). Depth contours of 200- and 2000-m are indicated by thin black lines. Note that color scales are different in each panel. Created with Matlab R2018B (www.mathworks.com).

Figure S 11. Seasonal average model-EKE (color shading) for a) Summer (Jan - Feb - Mar) over seasonal average model surface velocity (black arrows) and February cLCS (white lines). In b) seasonal average model-EKE (color shading) for Winter (Jul - Aug - Sep) over seasonal average model surface velocity (black arrows) and over August cLCS (white lines). In c) seasonal average model-MKE (color shading) for Summer (Jan - Feb - Mar) over seasonal average model surface velocity (black arrows) and February cLCS (white lines). In d) seasonal average model-MKE (color shading) for Winter (Jul - Aug - Sep) over seasonal average model surface velocity (black arrows) and August cLCS (white lines). In e) seasonal average model-TKE (color shading) for Summer (Jan - Feb - Mar) over seasonal average model surface velocity (black arrows) and February cLCS (white lines). In e) seasonal average model-TKE (color shading) for Winter (Jul - Aug - Sep) over seasonal average model surface velocity (black arrows) and August cLCS (white lines). Depth contours of 200- and 2000-m are indicated by thin black lines. Created with Matlab R2018B (www.mathworks.com).

7 Eulerian and Lagrangian divergence

Figure S 12. Monthly average of the Eulerian divergence (1/s) computed from the daily climatological velocity. Depth contours of 200- and 2000-m are indicated by thin black lines. Created with Matlab R2018B (www.mathworks.com).

Let δ be the Eulerian divergence, we can use the Lagrangian equation for area change given by $\mathrm{d}A(t)/\mathrm{d}t = \delta(t)A(t)$, where $A(t)$ is the time-dependant material area, to quantify along-path changes in area. Given an initial area A_0, the solution is:

$$A(t) = A_0 \exp\left(\int_{t_0}^{t} \delta\left(\mathbf{F}_{t_0}^{t'}(\mathbf{x}_0), t'\right) \mathrm{d}t'\right) \tag{1}$$

where $\delta\left(\mathbf{F}_{t_0}^{t'}(\mathbf{x}_0), t'\right)$ is the Eulerian divergence evaluated along the trajectories of the same flow maps $\mathbf{F}$, used to compute cLCS. From equation 1, we can define a fractional change of area as $\alpha := A(t)/A_0$. Note that α is the Jacobian determinant of the flow map, that is $\alpha = \det\left(\mathrm{D}\mathbf{F}_{t_0}^{t}\right)$. Thus we can quantify the effect of along-path divergence. For example, if α is equal to one, then there is no change of area, if $\alpha = 0.99$, then there is a 1% area decrease, and if $\alpha = 1.01$ there is a 1% area increase. In each month, the influence of divergence on persistent Lagrangian transport patterns is negligible, with mean area-change values of 0.01% and a standard deviation always less than 1% (Fig. 13).

Figure S 13. Monthly average of $\alpha - 1$ fields, obtained from the flow maps of the twelve dynamical systems used to compute cLCS in each month. A value of 0.01 means a 1% along-path area increase, and a value of -0.01 means a 1% along-path area decrease. Depth contours of 200- and 2000-m are indicated by thin black lines. Created with Matlab R2018B (www.mathworks.com).
