# Supplementary figures and images for: Publisher Correction: Persistent meanders and eddies lead to quasi‑steady Lagrangian transport patterns in a weak western boundary current
Source: Sci Rep. 2021 May 6;11:10115. doi: 10.1038/s41598-021-89612-7 (PMC8102625; doi:10.1038/s41598-021-89612-7)

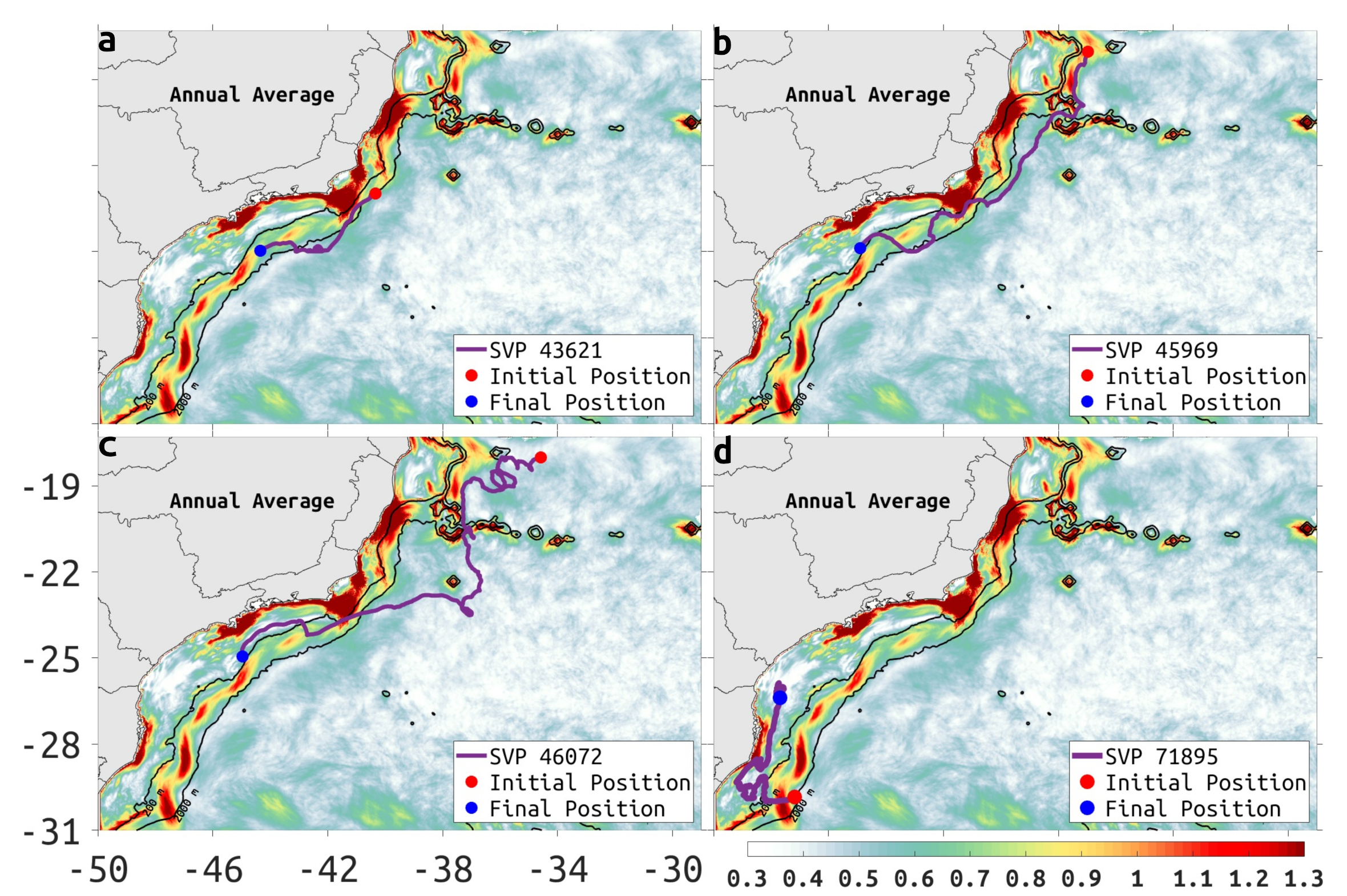

Supplement: Supplementary file 1 — Supplementary Figure 1. [file 41598_2021_89612_MOESM1_ESM.jpg]

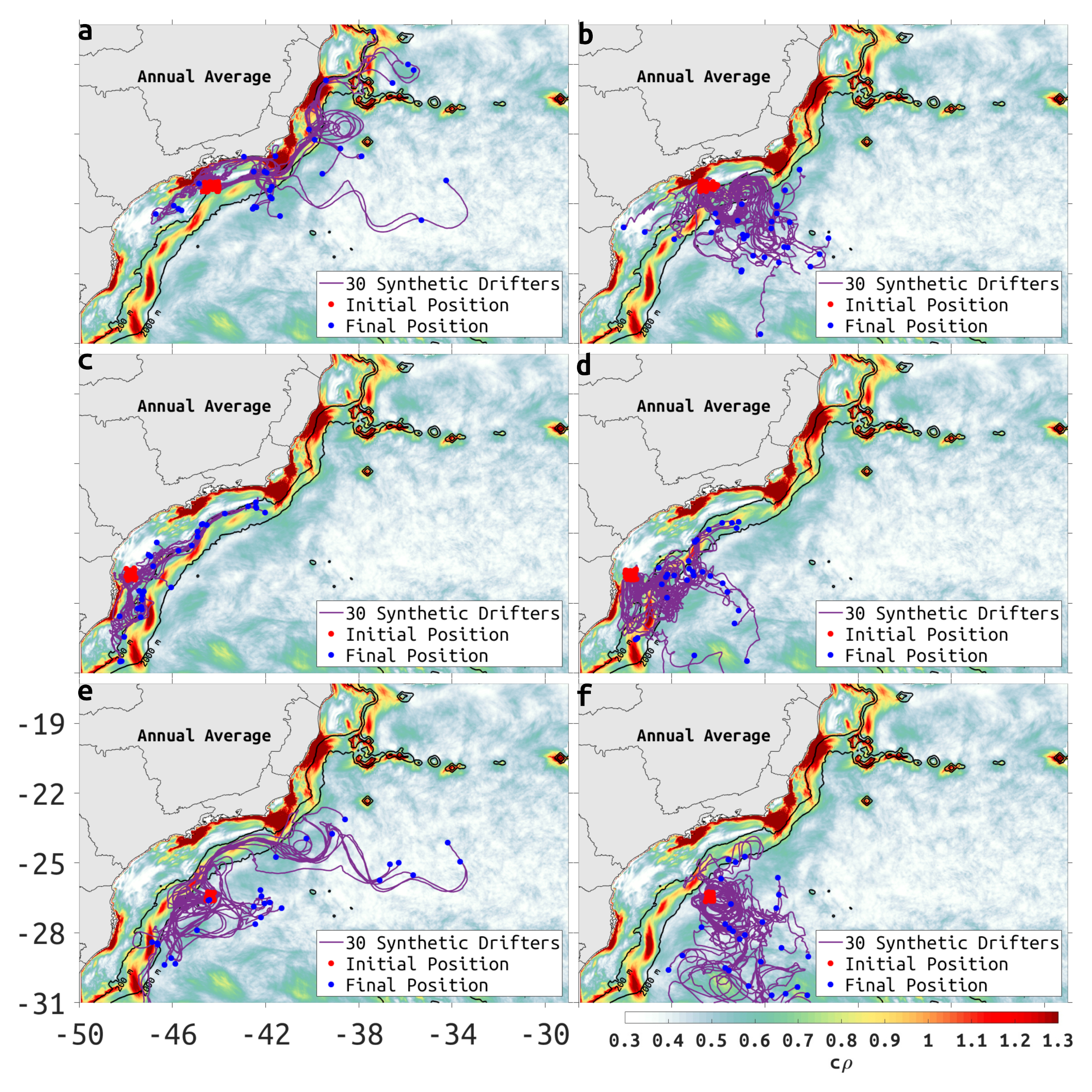

Supplement: Supplementary file 2 — Supplementary Figure 2. [file 41598_2021_89612_MOESM2_ESM.jpg]

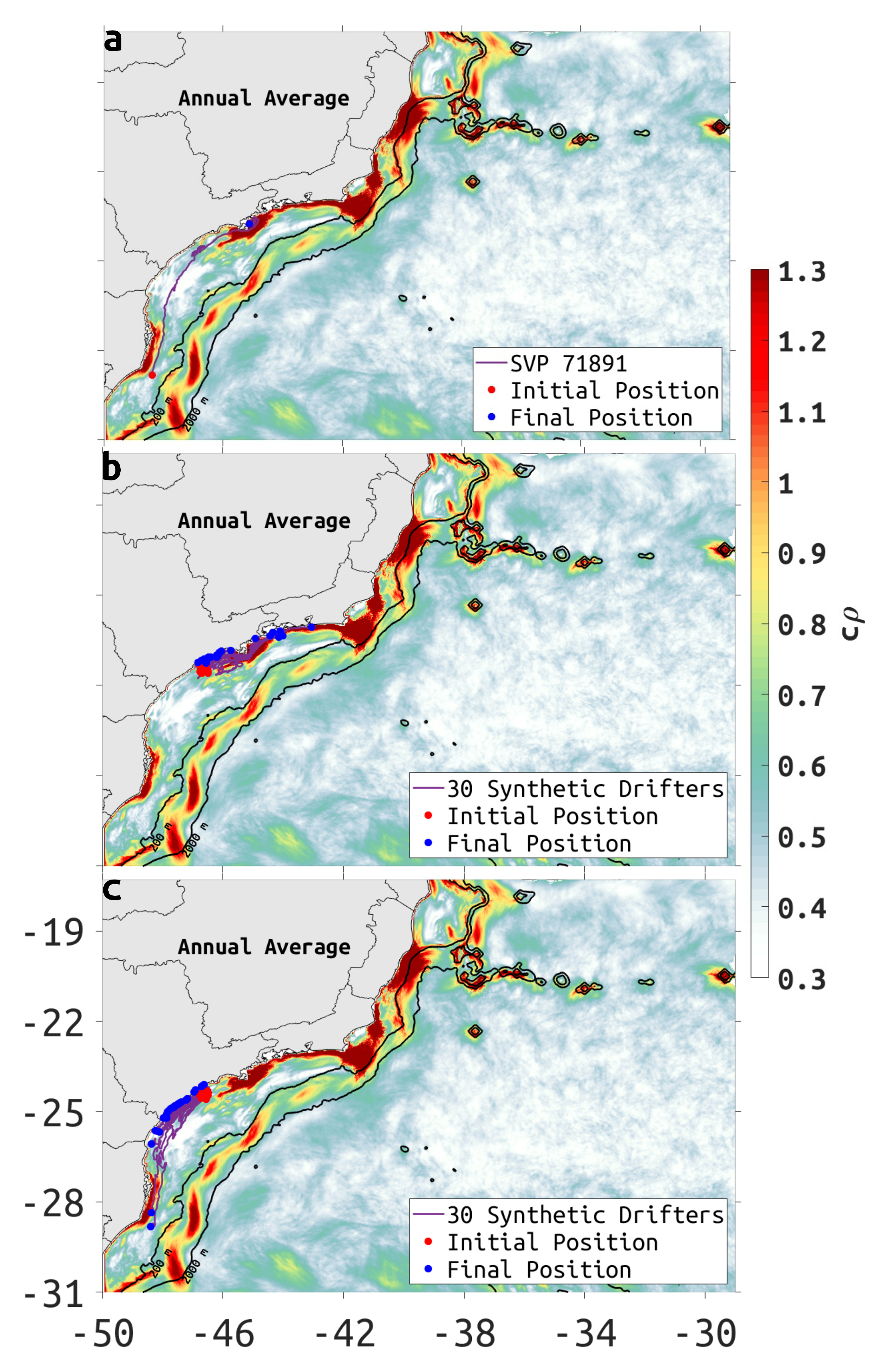

Supplement: Supplementary file 3 — Supplementary Figure 3. [file 41598_2021_89612_MOESM3_ESM.jpg]

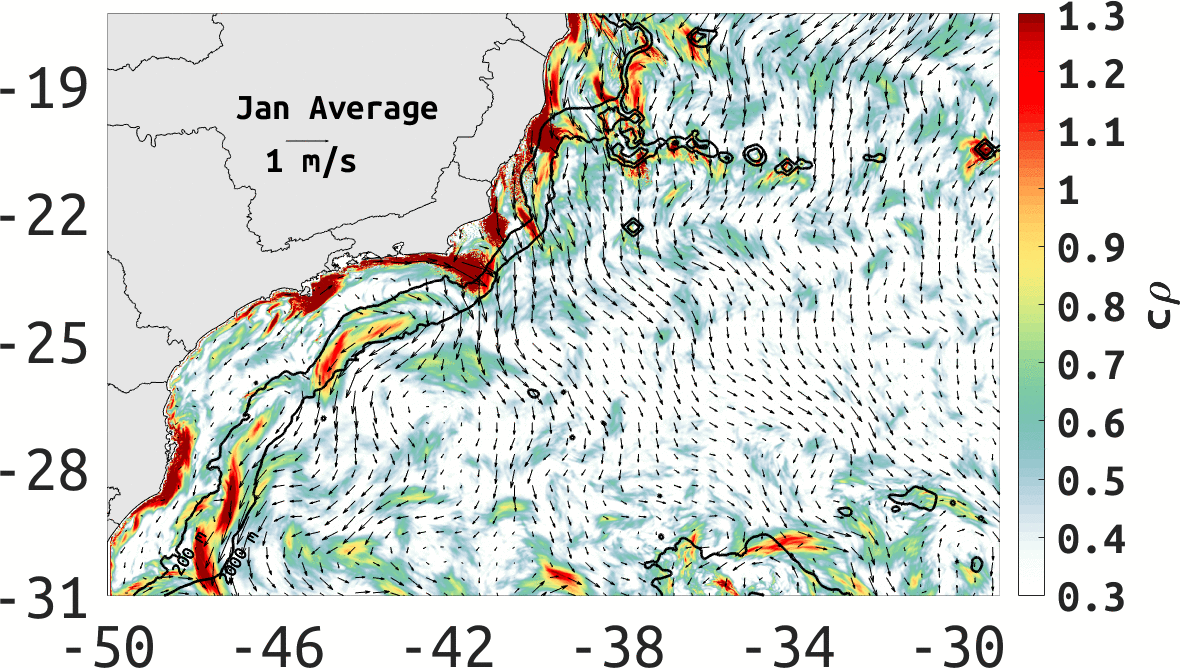

Supplement: Supplementary file 4 — Supplementary Figure 4. [file 41598_2021_89612_MOESM4_ESM.gif]

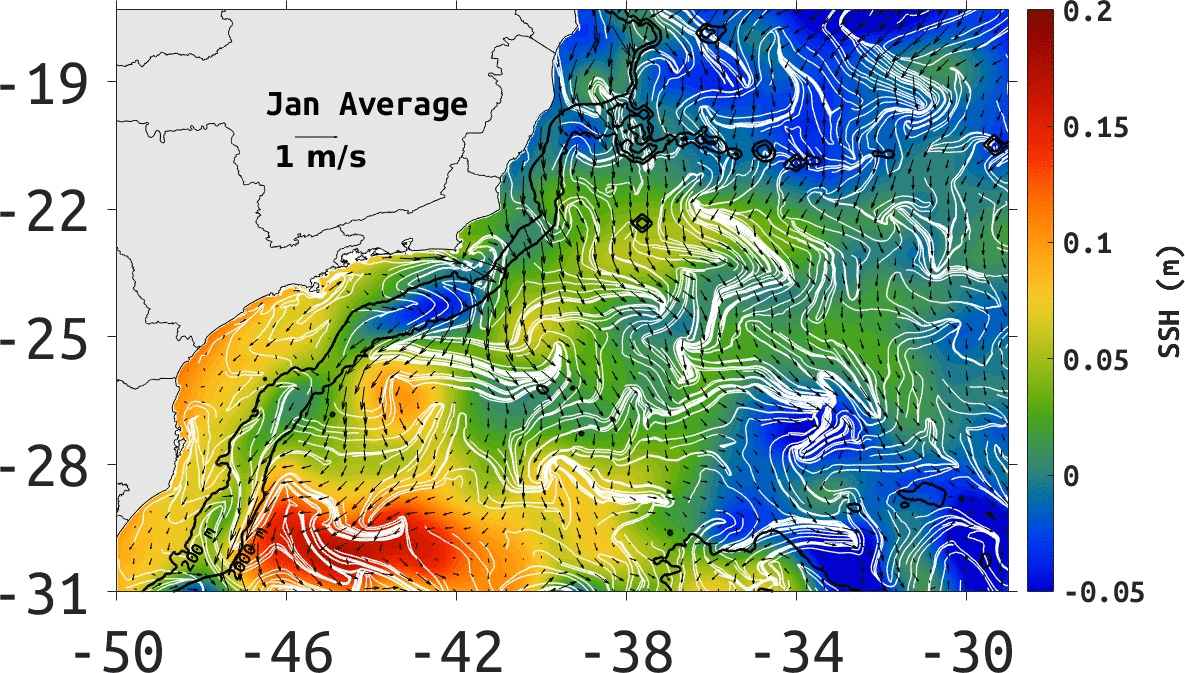

Supplement: Supplementary file 5 — Supplementary Figure 5. [file 41598_2021_89612_MOESM5_ESM.gif]

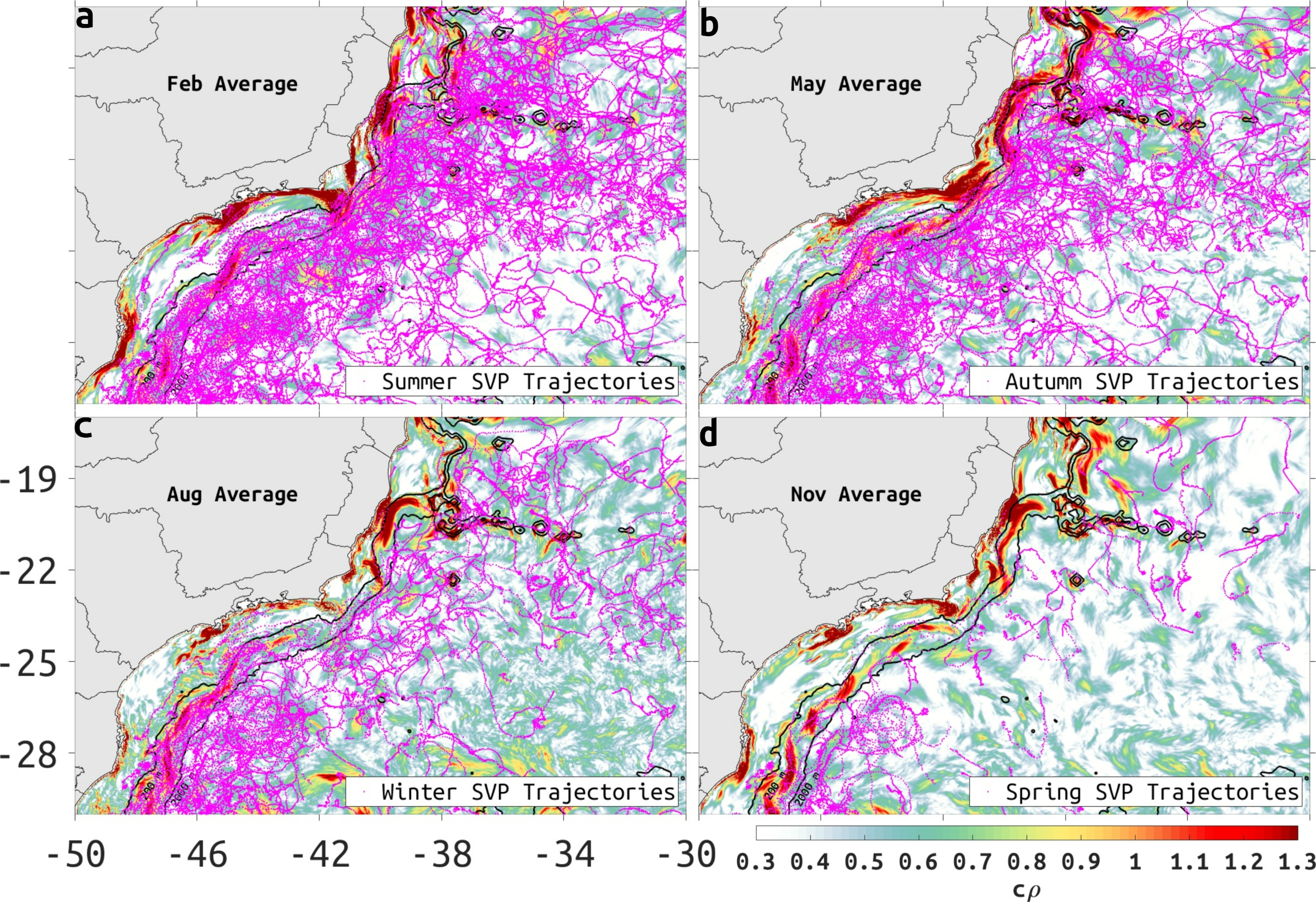

Supplement: Supplementary file 6 — Supplementary Figure 6. [file 41598_2021_89612_MOESM6_ESM.jpg]

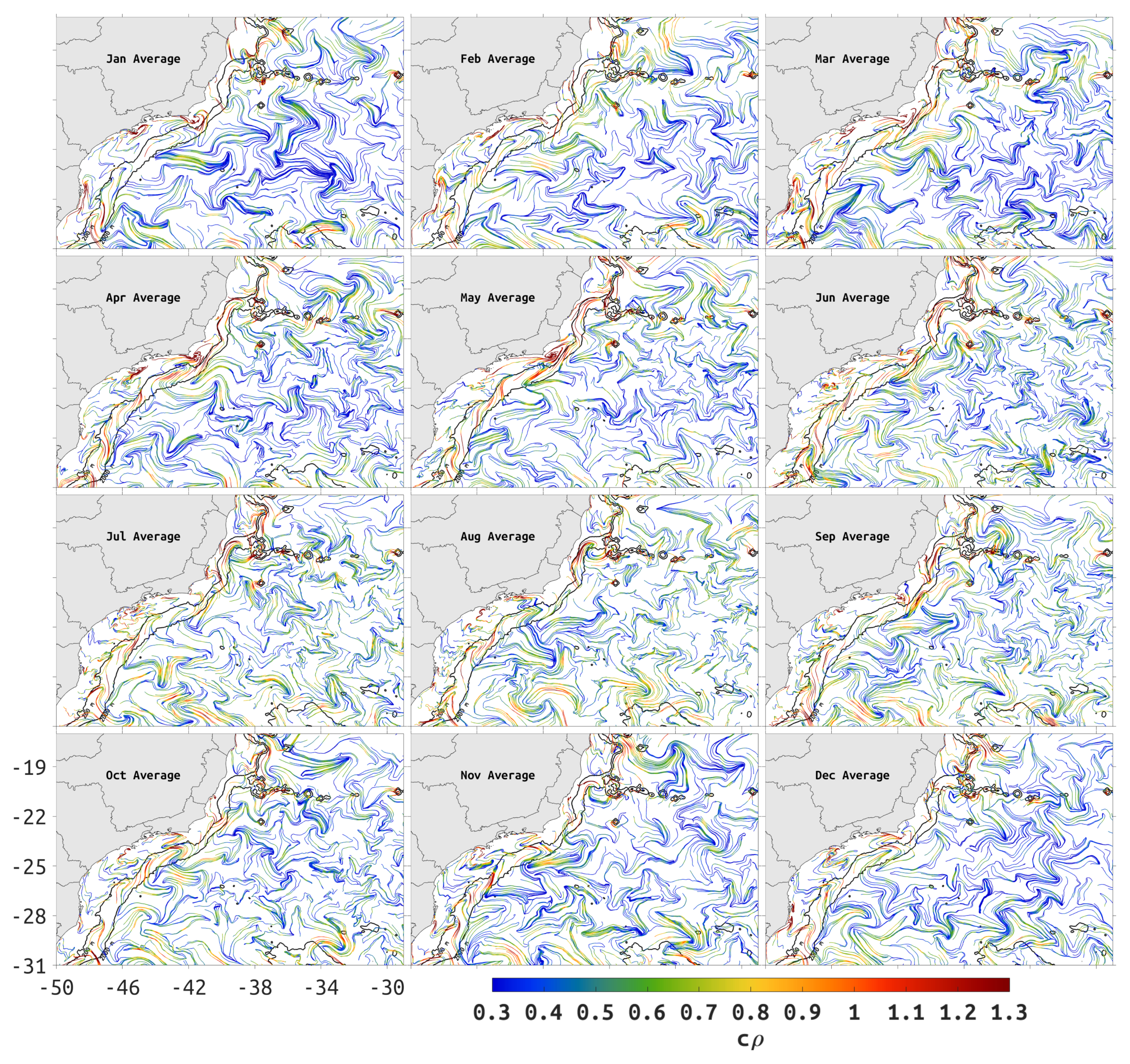

Supplement: Supplementary file 7 — Supplementary Figure 7. [file 41598_2021_89612_MOESM7_ESM.jpg]

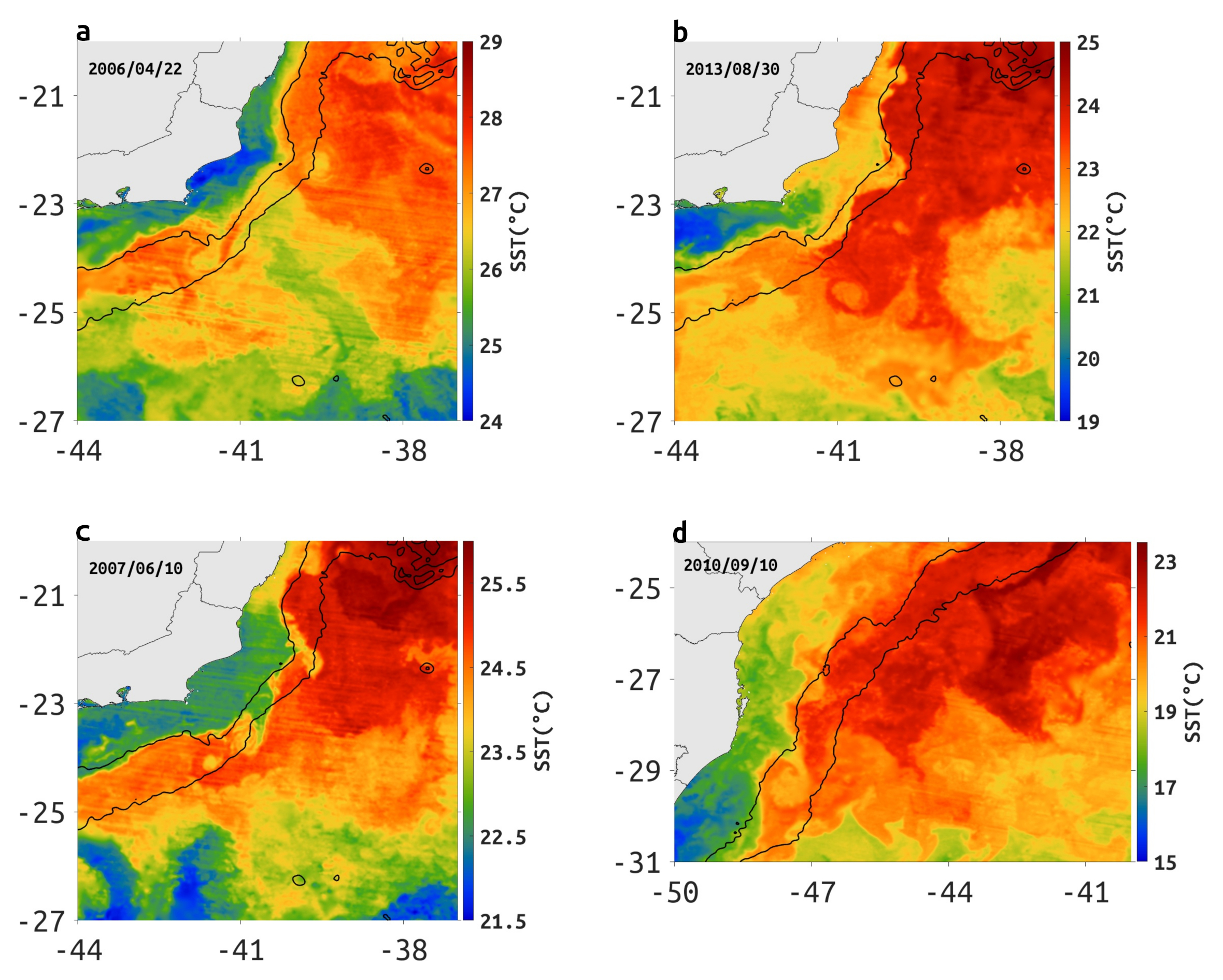

Supplement: Supplementary file 8 — Supplementary Figure 8. [file 41598_2021_89612_MOESM8_ESM.jpg]

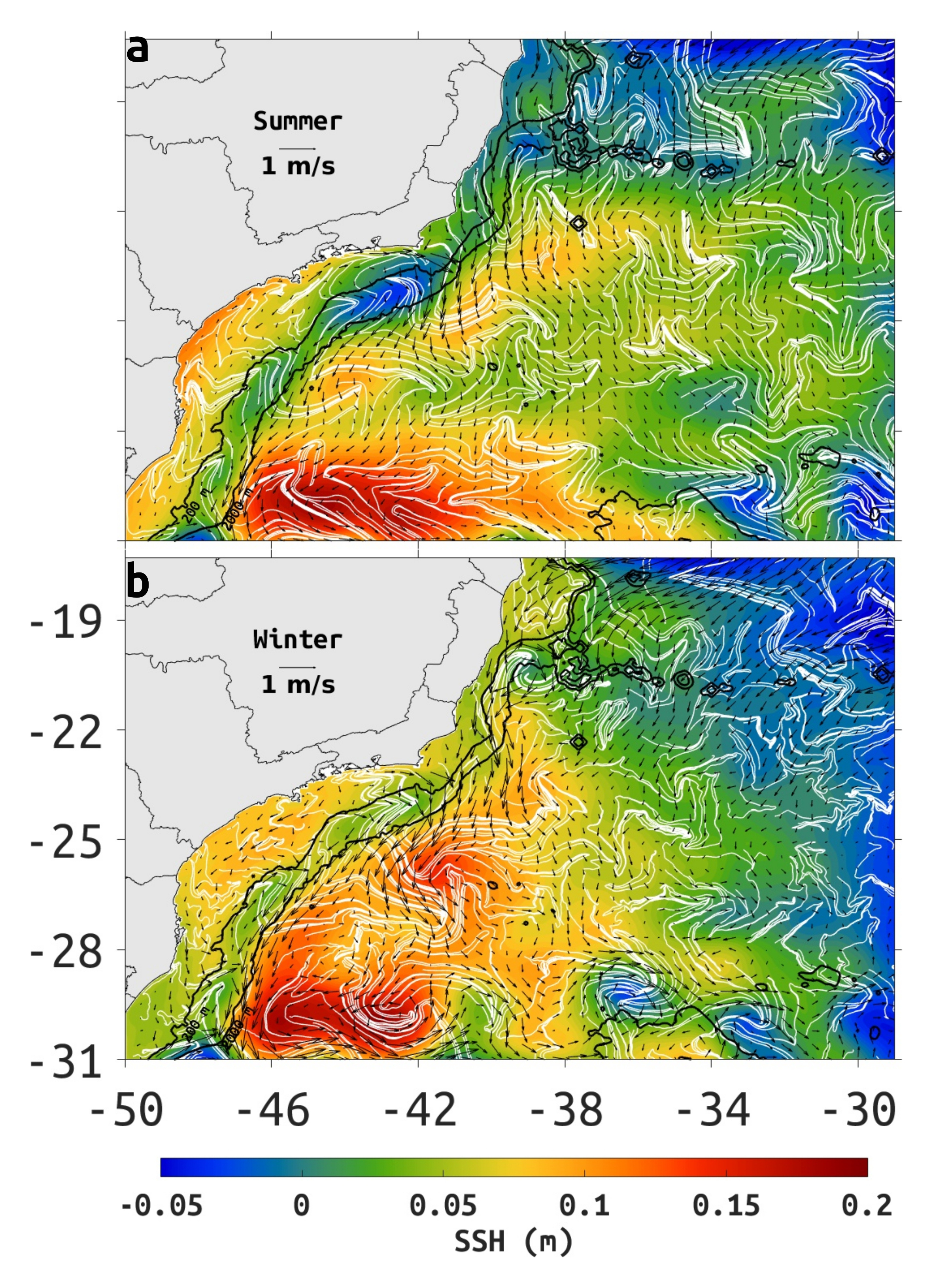

Supplement: Supplementary file 9 — Supplementary Figure 9. [file 41598_2021_89612_MOESM9_ESM.jpg]

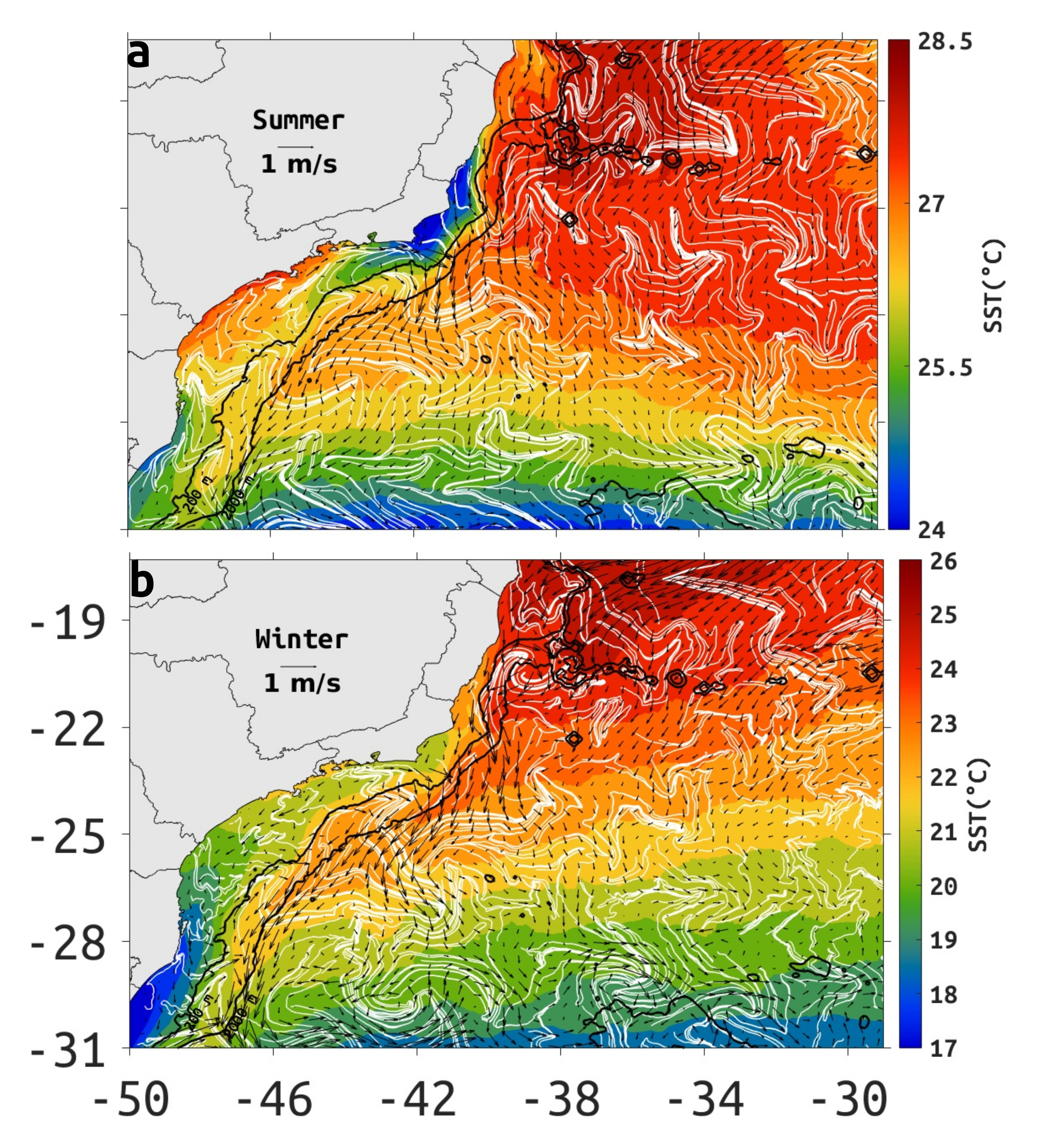

Supplement: Supplementary file 10 — Supplementary Figure 10. [file 41598_2021_89612_MOESM10_ESM.jpg]

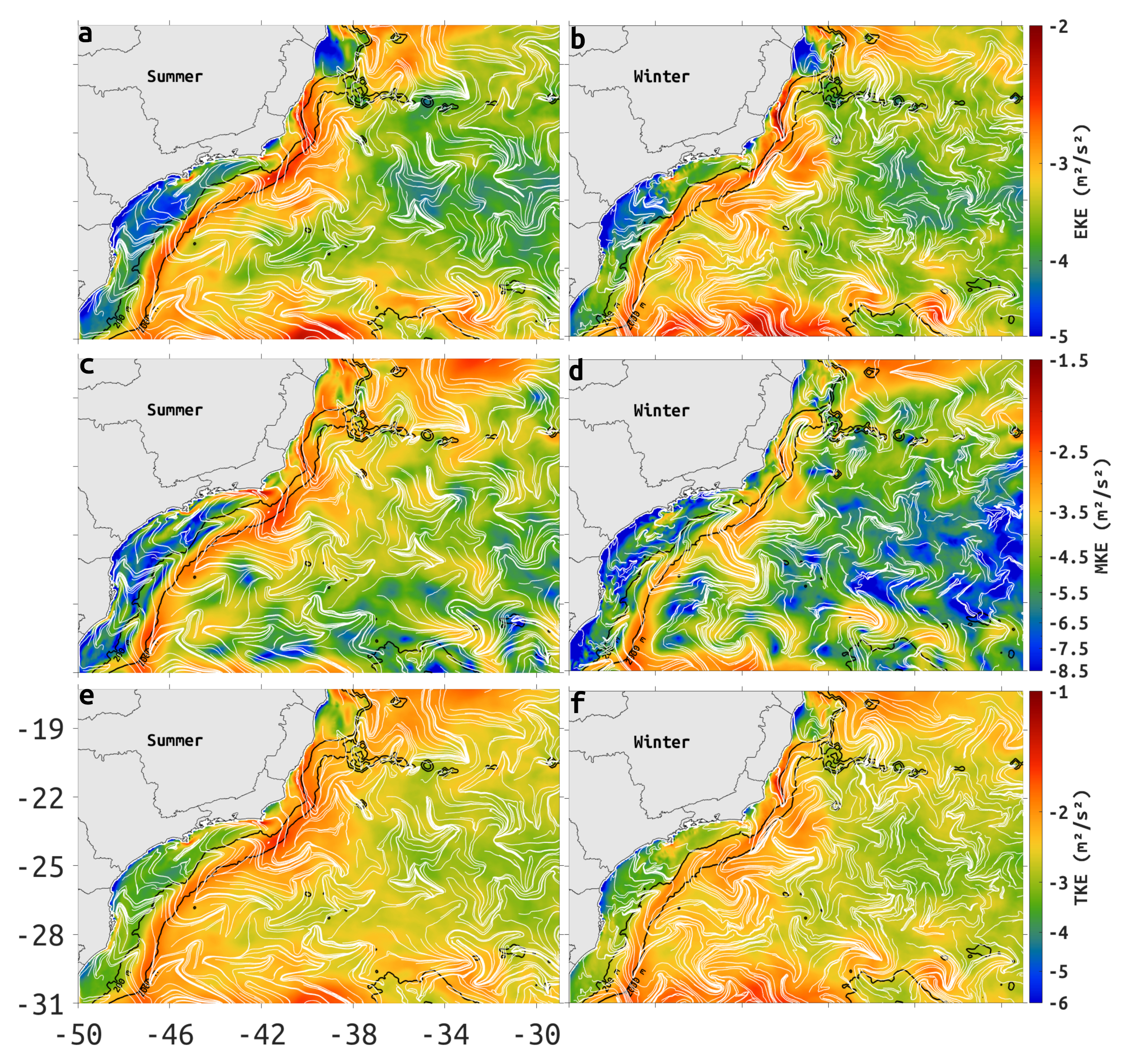

Supplement: Supplementary file 11 — Supplementary Figure 11. [file 41598_2021_89612_MOESM11_ESM.jpg]

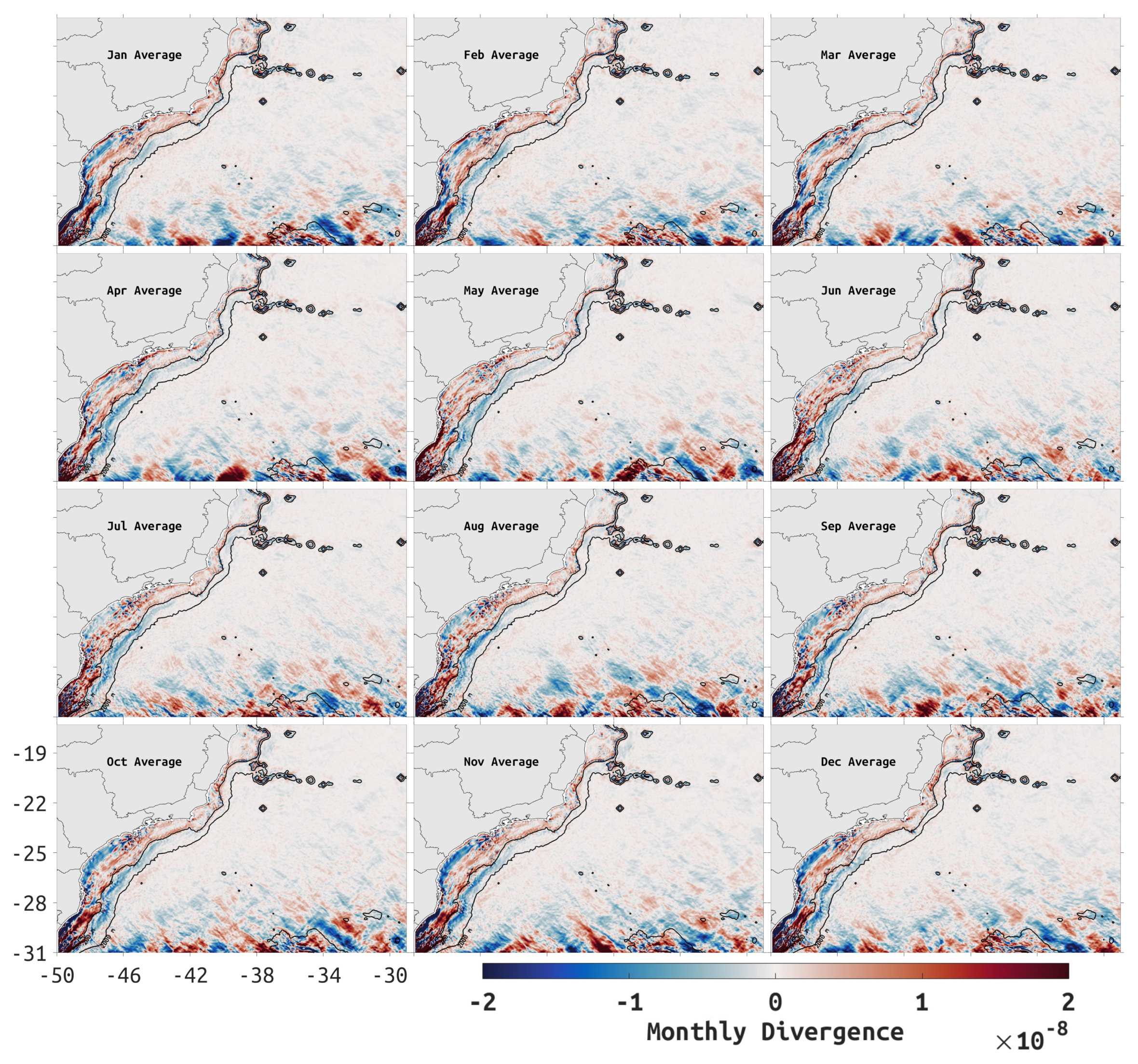

Supplement: Supplementary file 12 — Supplementary Figure 12. [file 41598_2021_89612_MOESM12_ESM.jpg]

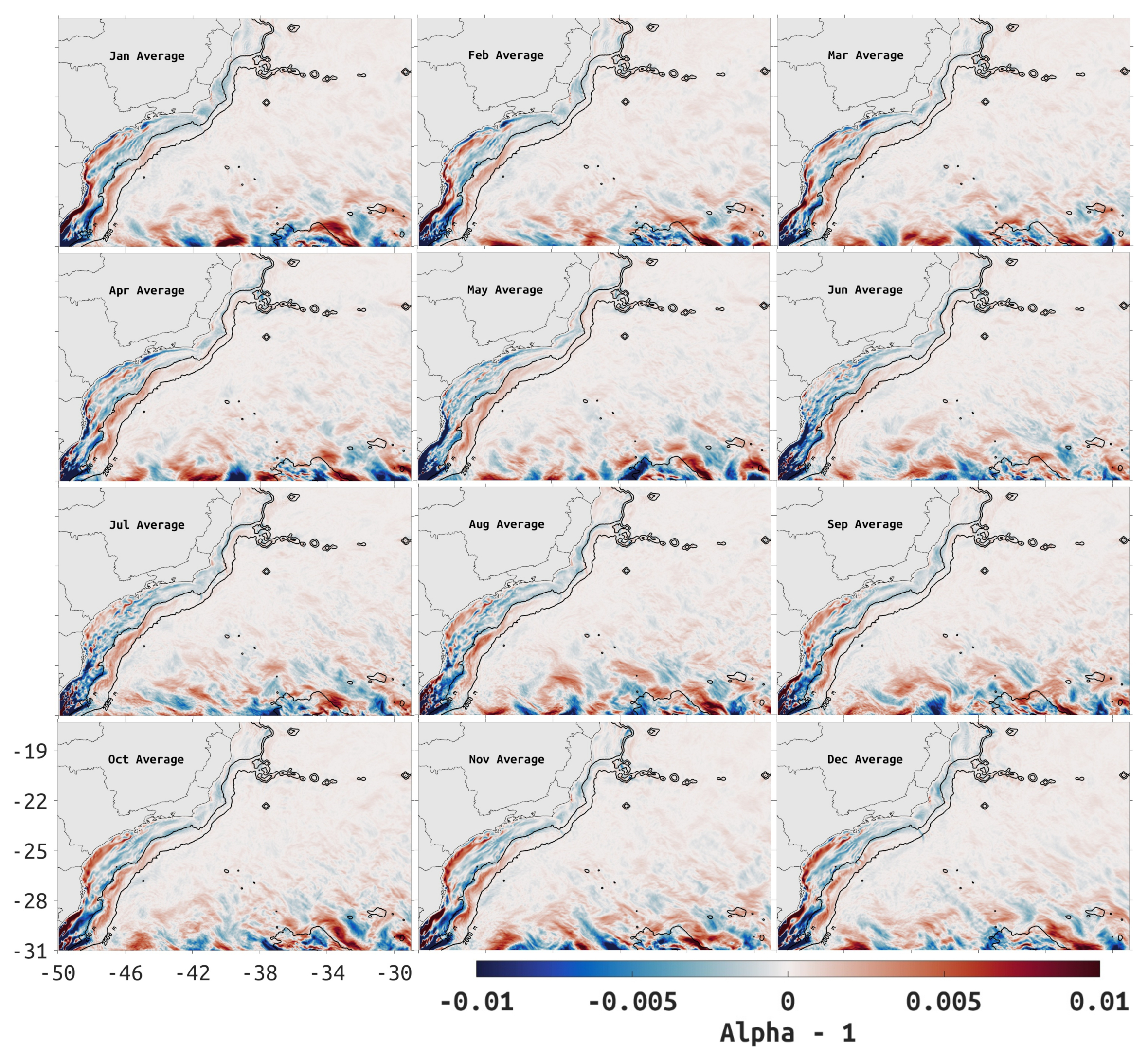

Supplement: Supplementary file 13 — Supplementary Figure 13. [file 41598_2021_89612_MOESM13_ESM.jpg]
